# Criteria-led discharge in paediatric inpatient care in Switzerland: a quality improvement study of implementation, safety and satisfaction

**DOI:** 10.1007/s00431-026-07578-y

**Published:** 2026-11-07

**Authors:** Mirjam Kissling, Anna Schoeni, Sibylle Jung, Karin Roth, Birgit Wernz, Marco Lurà, Nicole Ritz

**Affiliations:** 1 Center for Child Health Analytics (CCHA), Children’s Hospital of Central Switzerland, Lucerne, Switzerland; 2 Faculty of Health Sciences and Medicine, University of Lucerne, Lucerne, Switzerland; 3 Department of Paediatrics, Children’s Hospital of Central Switzerland, Lucerne, Switzerland; 4 University Children’s Hospital Basel and Department of Clinical Research University of Basel, Mycobacterial and Migrant Health Research Group, Basel, Switzerland; 5 Department of Research, Swiss Tropical and Public Health Institute, Allschwil, Switzerland

**Keywords:** Respiratory disease, Criteria-led discharge, Nurse-led discharge, Paediatrics

## Abstract

Paediatric hospitals have high admission rates and short lengths of stay, leading to a high turnover. The discharge process is challenged by delays and a lack of standardisation. Criteria-led discharge (CLD) has been proposed to overcome these challenges. CLD enables nursing staff to safely discharge patients once predefined clinical criteria are met, reducing reliance on physician review and minimising delays. We introduced CLD on a paediatric ward and evaluated its implementation in a quality improvement study pre- (December 2023–March 2024) and post-implementation (December 2024–March 2025). Outcomes were assessed using retrospective electronic health record data and surveys. The primary outcome was patient safety, measured by 14-day readmission and complication rates. Secondary outcomes included efficiency and patient flow, measured by length of stay, time of day at discharge and discharge delays, as well as patient, caregiver and staff satisfaction. A total of 355 eligible admissions (202 pre-, 153 post-implementation) were included into the analysis in three diagnostic groups: 122 bronchiolitis, 135 pneumonia and 98 asthma. 14-day readmission rates were comparable between CLD (3.2%), standard discharge (SD) (3.4%) and post-implementation SD (4.5%) cohorts. Patient, caregiver and staff satisfaction was high.

*Conclusions*: CLD is non-inferior in terms of safety compared to standard discharge. It subjectively improved discharge efficiency and positively impacted workload distribution while maintaining high patient, caregiver and staff satisfaction. CLD is a promising tool to facilitate discharges in paediatrics. Local adaptation and quality control are necessary for successful implementation.

**Table:** 

**What is Known:** • *Criteria-led discharge (CLD) increases standardisation, safety and efficiency in discharges from hospital.*
**What is New:** •* CLD is a safe method to discharge children from inpatient paediatric wards.* • *CLD may decrease stress of ward staff and enhance efficiency in paediatric hospitals.*

**Supplementary Information:**

The online version contains supplementary material available at https://doi.org/10.1007/s00431-026-07578-y.

## Introduction

Paediatric hospitals require highly efficient discharge management to respond to high patient turnover and winter surges combined with economic and staffing constraints. Discharge delays result from conservative or non-standardised management [1] and necessary physician review before discharge [2]. Criteria-led discharge (CLD) is a possible solution to these challenges. CLD enables a wider team and particularly nursing staff to safely discharge patients once predefined criteria are met, thus promoting efficient and standardised discharges [2–6].

Prior to our intervention, no paediatric hospital in Switzerland had implemented CLD, leaving its safety and acceptance in the local context unexplored. Internationally, only few reports describe paediatric CLD including standardised discharge criteria and delegation of the discharge decision to nursing staff [7–11]. While these suggest high safety, evidence in general non-surgical inpatient paediatric care remains scarce, with only few reports on children with asthma or wheezing conditions [8, 9]. Moreover, patient-reported outcomes such as acceptance of and satisfaction with CLD [12], as well as interprofessional staff perspectives regarding the shift of responsibilities or satisfaction in paediatric settings, are largely unknown. With this quality improvement study, we aimed to assess safety, efficiency and satisfaction with CLD in paediatrics.

## Methods

We report on 4 months of CLD introduction using the Standards for Quality Improvement Reporting Excellence (SQUIRE) 2.0 guidelines’ framework [13].

### Context

Our site of intervention, the Children’s hospital of Central Switzerland, is a 96-bed, tertiary teaching hospital. The intervention ward is a 27-bed inpatient paediatric ward. The clinical providers on the ward, including part-time staff, consist of 76 nurses (41 certified nurses, 4 healthcare assistants, 31 students), 4 resident physicians on rotations from a team of 32 and a core team of 5 consultants, with 20 further specialist consultants supporting during weekends. Before the start of implementation, the immunisation with nirsevimab for respiratory syncytial virus (RSV) became available in Switzerland in October 2024 [14].

### Intervention

#### Project preparation

A project team consisting of the head of department, a senior physician and representatives of the nursing staff selected diagnoses suitable for CLD based on prevalence and predictability of the disease. Bronchiolitis, pneumonia and asthma/obstructive bronchitis were chosen for initial implementation. Discharge criteria were defined incorporating available information from published guidelines and where necessary reviewed by specialists. Criteria include physical condition, treatment milestones and organised aftercare. All staff were trained on CLD before implementation.

#### Implementation

**Fig. 1:**
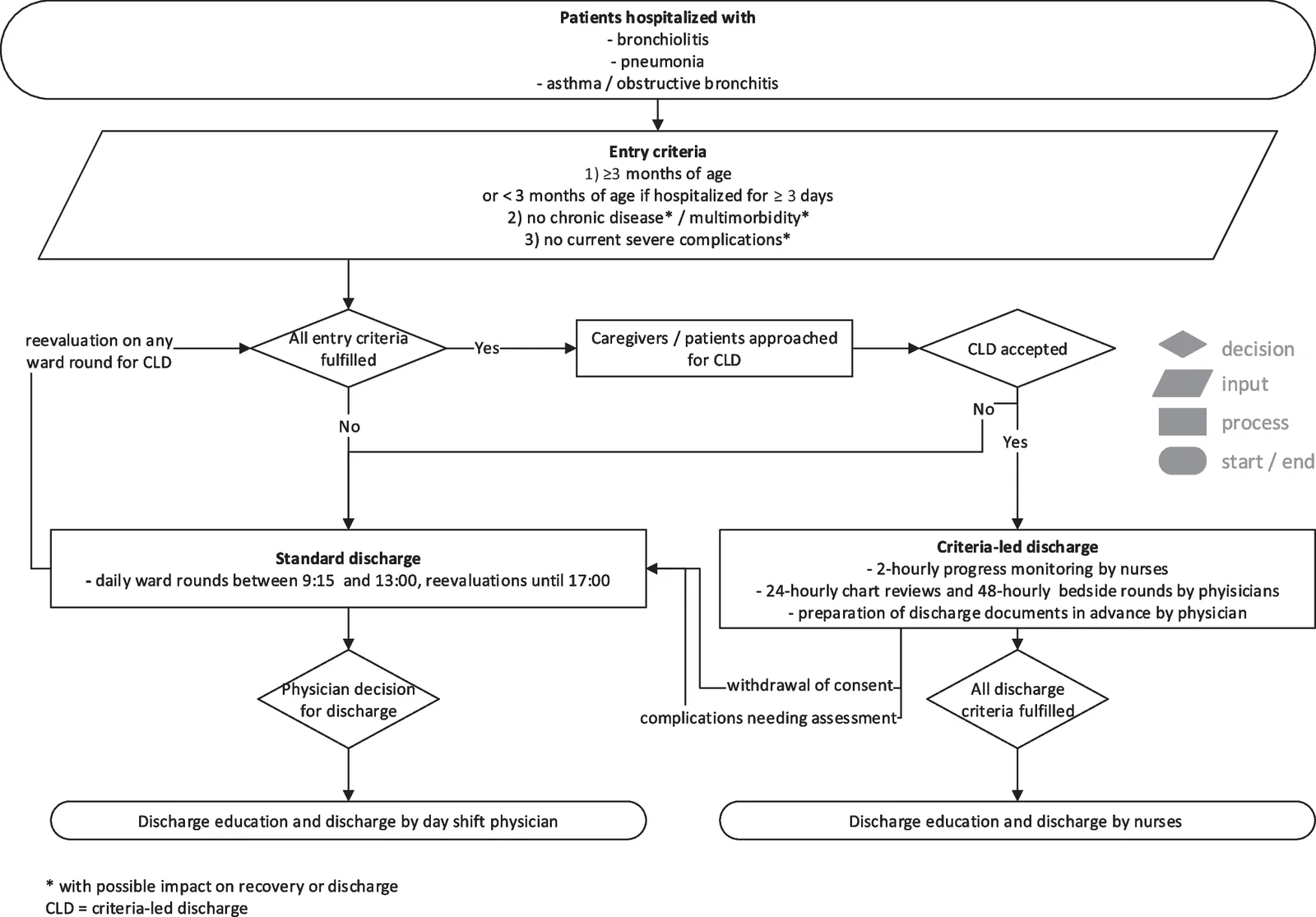
Flowchart showing patient selection and discharge pathways of criteria-led discharge and standard discharge

Implementation of CLD started on December 2, 2024. Patient selection and discharge pathway are displayed in Fig. 1. Children < 3 months were only considered eligible for CLD after a minimum duration of hospitalisation of 3 days, accounting for increased risk for secondary deterioration after discharge if included early in the disease course [15]. Physicians were responsible for selecting patients and obtaining consent for CLD. All consented patients were assigned the disease-specific catalogue of discharge criteria (supplemental Box S1).

### Study of the intervention

Outcomes included (1) patient safety including readmissions or reassessments, complications and mortality; (2) patient flow metrics including length of stay, discharge timing and delay of discharge; (3) adherence to discharge criteria; and (4) patient/caregiver and staff satisfaction.

### Measures

#### Data collection

Anonymised data on safety and patient flow were retrospectively collected from routine data captured in the electronic health record (EHR; Epic Systems Corporation). We included admissions with discharge from 02.12.2024 to 31.03.2025 and a comparative pre-implementation cohort discharged from 02.12.2023 to 31.03.2024. Information extracted comprised patient characteristics, diagnoses, admission and discharge information, laboratory/radiology results, clinical assessments, supportive therapies and medications administered (supplemental Box S2). Data on satisfaction was collected in anonymous and voluntary surveys via Microsoft forms.

#### Cohort definition

Admissions were grouped into pre-implementation (winter 2023/24) and post-implementation (winter 2024/25) cohorts and further classified by discharge type into CLD and SD cohorts. Patients with CLD-discontinuation were excluded from the main cohorts and described separately. Fast discharges, defined as admissions between 18:00 and 08:00 with same day discharge before 14:00, were excluded from analysis, as CLD would not have been initiated in this setting.

All admissions were manually reviewed to determine eligibility and assign diagnostic groups. Patients with relevant complications were considered non-eligible. Eligible admissions were assigned to the diagnostic groups of bronchiolitis, pneumonia and asthma/obstructive bronchitis (hereafter “asthma”). The criteria used for group assignment are detailed in supplemental Box S3*.* An upper age limit of 2 years was applied to the bronchiolitis diagnostic group, in accordance with international guidelines [16, 17].

#### Outcome measurements

The primary outcome was safety of CLD, assessed by 14-day readmission rates, reassessment rates in the emergency department, complication rates and mortality. All readmissions and reassessments post-implementation were chart-reviewed. Complications were assessed through review of text diagnoses. Acute comorbidities or complications not leading to ineligibility were recorded and classified as “minor”. Mortality was assessed both on the intervention ward and other wards, to capture adverse outcomes amongst patients who had been transferred. The secondary outcomes of patient flow metrics included the time of day at discharge, length of inpatient stay and timespan between the end of supplemental oxygen requirement to discharge.

Both safety and patient flow metrics were analysed stratified by diagnostic group, accounting for population differences. Further, adherence to objectively traceable discharge criteria was assessed as a secondary outcome (details in supplemental Box S4).

Patient/caregiver satisfaction was assessed at discharge using a survey distributed to those approached for CLD. The survey included 5-point bipolar Likert scale items evaluating the perceived adequacy of discharge timing, quality of discharge-related communication, knowledge of discharge criteria, remaining questions at discharge and sense of safety when returning home.

Staff satisfaction (nurses and physicians) was assessed via surveys at baseline (December 2024) and follow-up (March 2025). At both timepoints, staff assessed workday satisfaction, perceived stress, workload, interprofessional communication, discharge efficiency and reasons for discharge delay. Furthermore, general attitudes towards CLD were assessed at baseline. At follow-up, staff evaluated whether observed changes were attributable to CLD and whether CLD should be maintained. Nurses additionally reported their views on acquired competencies. Most items used 5-point bipolar Likert scales with neutral options. Reasons for discharge delay were queried via multiple choice format.

#### Analysis

The primary (safety) outcomes were analysed for CLD compared to SD post-implementation as well as to the pooled SD cohorts (pre- and post-implementation). The secondary outcomes of patient flow metrics were compared between pre- and post-implementation cohorts and between CLD and SD post-implementation.

To assess the impact of CLD versus SD on length of stay post-implementation, we used a multivariable linear regression model adjusted for relevant covariates (supplemental Box S5). Further, among post-implementation patients requiring supplemental oxygen, we assessed the impact of CLD versus SD on time to discharge after oxygen discontinuation using a multivariable linear regression model adjusted for relevant covariates (supplemental Box S6).

We used descriptive statistics to summarise baseline and outcome characteristics. Group comparisons of normally distributed continuous variables were done using the unpaired t-test and of non-normally distributed variables using the Mann–Whitney *U* test. Categorical variables were compared using Fisher’s exact test (for *n* < 5) and *ꭓ*^2^-test (for *n* ≥ 5).

Likert scale items of staff surveys were compared between timepoints using Kruskal–Wallis test and Dunn-Bonferroni post hoc analysis. Analyses were stratified into professional subgroups, whereas junior and senior physicians as well as all academic levels of nurses were grouped together. The patient/caregiver survey was grouped descriptively by discharge type.

All analyses were performed using R version 4.4.0, with a significance level of 0.05 and two-tailed tests.

## Results

### Participants

We identified 521 admissions (279 pre-, 242 post-implementation) with the prespecified diagnoses and discharge during the periods of interest. Of these, 166 were excluded as non-eligible for CLD due to patient’s chronic disease, current comorbidities or complications, young age or classification as fast discharges (supplemental Fig. S1). Complications and comorbidities leading to exclusion are described in supplemental Fig. S2. A total of 355 eligible admissions (202 pre-, 153 post-implementation) were included into the analysis in the three diagnostic groups: 122 bronchiolitis, 135 pneumonia and 98 asthma. Of the eligible post-implementation admissions, 40.5% (*n* = 62) were in the CLD and 57.5% (*n* = 88) in the SD cohort. CLD was discontinued in 2.0% (*n* = 3) due to withdrawal of consent, need for further investigations or unspecified reasons. These were excluded from comparative analysis. Baseline characteristics are presented in Tables 1 and 2.

**Table 1:** Baseline characteristics, stratified by diagnostic group and pre- and post-implementation cohort

	Overall	All diagnoses		*p*-value	Bronchiolitis		*p*-value	Pneumonia		*p*-value	Asthma/obstructive bronchitis		*p*-value
Number of cases	352	352			120			134			98		
CLD implementation period	All	Pre	Post		Pre	Post		Pre	Post		Pre	Post	
**Number**	**352**	**202**	**150**		**79**	**41**		**67**	**67**		**56**	**42**	
Female, *n* (%)	168 (47.7)	91 (45.0)	77 (51.3)	0.29	36 (45.6)	19 (46.3)	1	30 (44.8)	35 (52.2)	0.49	25 (44.6)	23 (54.8)	0.43
Age in months, median [IQR]	18.1 [6.9, 44.5]	15.4 [4.5, 41.1]	22.9 [12.0, 53.3]	< 0.001	3.8 [2.4, 6.9]	8.4 [5.3, 12.6]	< 0.001	35.8 [18.3, 63.4]	39.2 [17.3, 67.1]	0.71	29.6 [16.5, 52.0]	38.5 [20.4, 59.6]	0.25
Cases with known prematurity, *n* (%)	40 (11.4)	25 (12.4)	15 (10.0)	0.60	10 (12.7)	5 (12.2)	1	6 (9.0)	3 (4.5)	0.49	9 (16.1)	7 (16.7)	1
ICU stay, *n* (%)	22 (6.2)	12 (5.9)	10 (6.7)	0.96	6 (7.6)	5 (12.2)	0.62	5 (7.5)	2 (3.0)	0.44	1 (1.8)	3 (7.1)	0.31
Nasogastric tube placed, *n* (%)	79 (22.4)	63 (31.2)	16 (10.7)	< 0.001	50 (63.3)	10 (24.4)	< 0.001	10 (14.9)	4 (6.0)	0.16	3 (5.4)	2 (4.8)	1
Cases with need of supplemental oxygen during hospitalisation, *n* (%)	302 (85.8)	182 (90.1)	120 (80.0)	0.01	71 (89.9)	33 (80.5)	0.25	57 (85.1)	52 (77.6)	0.38	54 (96.4)	35 (83.3)	0.04
Time interval of first to last oxygen supplementation in hours, median [IQR]^a^	33.8 [13.6, 66.1]	40.9 [15.7, 79.1]	27.0 [9.00, 47.0]	< 0.001	63.0 [21.8, 97.7]	30.8 [9.1, 66.8]	0.02	43.7 [23.1, 69.0]	30.5 [9.5, 50.4]	0.15	26.4 [10.1, 42.1]	25.9 [7.9, 31.8]	0.47
Swab results positive for any significant respiratory pathogen, *n* (%)^b^	220 (62.5)	130 (64.4)	90 (60.0)	0.47	67 (84.8)	32 (78.0)	0.50	50 (74.6)	43 (64.2)	0.26	13 (23.2)	15 (35.7)	0.26
RSV positivity, *n* (%)	178 (50.6)	110 (54.5)	68 (45.3)	0.11	67 (84.8)	31 (75.6)	0.32	31 (46.3)	24 (35.8)	0.29	12 (21.4)	13 (31.0)	0.40
Suspected bacterial infection, *n* (%)	75 (21.3)	35 (17.3)	40 (26.7)	0.047	0 (0.0)	0 (0.0)	NA	35 (52.2)	40 (59.7)	0.49	0 (0.0)	0 (0.0)	NA
CRP measured, *n* (%)	170 (48.3)	94 (46.5)	76 (50.7)	0.51	30 (38.0)	13 (31.7)	0.63	48 (71.6)	48 (71.6)	1	16 (28.6)	15 (35.7)	0.59
Highest CRP, median [IQR]	28.0 [9.0, 74.0]	22.0 [8.3, 60.5]	32.0 [12.8, 93.0]	0.048	11.1 [5.9, 23.8]	13.0 [8.0, 28.0]	0.55	57.5 [22.1, 104.3]	58.5 [22.5, 115.5]	0.73	10.5 [5.3, 16.6]	21.0 [12.0, 40.0]	0.03
Cases that received resp. antibiotics during hospitalisation, *n* (%)	98 (27.8)	50 (24.8)	48 (32.0)	0.17	5 (6.3)	2 (4.9)	1	42 (62.7)	44 (65.7)	0.86	3 (5.4)	2 (4.8)	1
Number of salbutamol inhalations per hospital day, median [IQR]; mean (SD)	1.3 [0.0, 6.2]	1.1 [0.0, 6.0]	1.5 [0.0, 6.3]	0.14	0.0 [0.0, 0.3]	0.8 [0.0, 2.1]	< 0.001	1.0 [0.0, 4.0]	0.4 [0.0, 1.5]	0.08	7.1 (1.7)	7.9 (2.8)	0.13
Antipyretic doses per hospital day, median [IQR]; mean (SD)	2.6 [0.9, 3.9]	2.9 [1.1, 4.0]	2.1 [0.9, 3.5]	0.06	3.4 (1.7)	3.4 (2.0)	0.89	2.9 [0.8, 4.0]	2.1 [0.9, 3.5]	0.31	1.7 [0.3, 2.6]	1.1 [0.1, 2.3]	0.42

^a^For cases with any oxygen supplementation

^b^Details available as supplemental Box S8

**Table 2:** Baseline characteristics of post-implementation cohort, stratified by diagnostic group and CLD and SD cohort

	Overall	All diagnoses		*p*-value	Bronchiolitis		*p*-value	Pneumonia		*p*-value	Asthma/obstructive bronchitis		*p*-value
Number of cases	150	150			41			67			42		
Method of discharge	All	CLD	SD		CLD	SD		CLD	SD		CLD	SD	
**Number**	**150**	**62**	**88**		**22**	**19**		**25**	**42**		**15**	**27**	
Female, *n* (%)	77 (51.3)	35 (56.5)	42 (47.7)	0.38	12 (54.5)	7 (36.8)	0.41	14 (56.0)	21 (50.0)	0.82	9 (60.0)	14 (51.9)	0.85
Age in months, median [IQR]	22.9 [12.0, 53.3]	22.8 [10.4, 55.6]	23.8 [12.9, 51.7]	0.58	7.5 [5.6, 11.6]	9.1 [4.0, 14.3]	0.81	34.4 [20.1, 70.0]	39.3 [15.6, 65.5]	0.42	39.5 [24.9, 63.3]	37.5 [17.7, 54.9]	0.68
Cases with known prematurity, *n* (%)	15 (10.0)	8 (12.9)	7 (8.0)	0.47	4 (18.2)	1 (5.3)	0.35	1 (4.0)	2 (4.8)	1	3 (20.0)	4 (14.8)	0.69
ICU stay, *n* (%)	10 (6.7)	4 (6.5)	6 (6.8)	1	3 (13.6)	2 (10.5)	1	1 (4.0)	1 (2.4)	1	0 (0.0)	3 (11.1)	0.54
Nasogastric tube placed, *n* (%)	16 (10.7)	9 (14.5)	7 (8.0)	0.31	6 (27.3)	4 (21.1)	0.73	2 (8.0)	2 (4.8)	0.63	1 (6.7)	1 (3.7)	1
Cases with need of supplemental oxygen during hospitalisation, *n* (%)	120 (80.0)	53 (85.5)	67 (76.1)	0.23	18 (81.8)	15 (78.9)	0.07	22 (88.0)	30 (71.4)	0.14	13 (86.7)	22 (81.5)	1
Time interval of first to last oxygen supplementation in hours, median [IQR]^a^	27.0 [9.0, 47.0]	33.9 [16.1, 59.8]	21.7 [6.9, 38.2]	0.005	40.7 [22.7, 82.3]	11.3 [5.6, 44.8]	0.07	35.9 [14.4, 49.0]	21.3 [8.1, 51.9]	0.30	28.6 [8.9, 40.7]	22.1 [6.7, 27.4]	0.12
Swab results positive for any significant respiratory pathogen, *n* (%)^b^	90 (60.0)	45 (72.6)	45 (51.1)	0.01	18 (81.8)	14 (73.7)	0.71	20 (80.0)	23 (54.8)	0.07	7 (46.7)	8 (29.6)	0.44
RSV positivity, *n* (%)	68 (45.3)	33 (53.2)	35 (39.8)	0.14	17 (77.3)	14 (73.7)	1	10 (40.0)	14 (33.3)	0.77	6 (40.0)	7 (25.9)	0.55
Suspected bacterial infection, *n* (%)	40 (26.7)	12 (19.4)	28 (31.8)	0.13	0 (0.0)	0 (0.0)	NA	12 (48.0)	28 (66.7)	0.21	0 (0.0)	0 (0.0)	NA
CRP measured, *n* (%)	76 (50.7)	32 (53.3)	44 (50.0)	0.98	4 (18.2)	9 (47.4)	0.09	20 (80.0)	28 (66.7)	0.37	8 (53.3)	7 (25.9)	0.15
Highest CRP, median [IQR]	32.0 [12.8, 93.0]	32.0 [19.0, 80.8]	37.5 [8.0, 100.0]	0.91	24.0 [18.3, 51.5]	8.0 [7.0, 25.0]	0.12	45.5 [19.3, 94.5]	63.0 [28.3, 139.0]	0.23	25.0 [19.0, 57.0]	20.0 [6.0, 24.5]	0.16
Cases that received resp. antibiotics during hospitalisation, *n* (%)	48 (32.0)	14 (22.6)	34 (38.6)	0.06	2 (9.1)	0 (0.0)	0.49	11 (44.0)	33 (78.6)	0.009	1 (6.7)	1 (3.7)	1
Number of salbutamol inhalations per hospital day, median [IQR]; mean (SD)	3.3 (3.8)	2.9 (3.6)	3.6 (4.0)	0.35	0.7 [0.0, 1.9]	1.2 [0.0, 4.0]	0.47	0.7 [0.0, 1.4]	0.4 [0.0, 1.5]	0.81	7.9 (2.6)	7.9 (3.0)	0.96
Antipyretic doses per hospital day, median [IQR]; mean (SD)	2.1 [0.9, 3.5]	2.5 [1.1, 3.9]	1.6 [0.7, 3.3]	0.16	3.1 (1.9)	3.6 (2.1)	0**.**41	2.4 [1.3, 3.6]	1.5 [0.6, 3.1]	0.16	1.1 [0.0, 3.0]	1.2 [0.5, 1.9]	0.86

^a^For cases with any oxygen supplementation

^b^Details available as supplemental Box S8

### Cohort description

Between pre- and post-implementation, a general increase of the median (IQR) age of patients from 15.4 (4.5, 41.1) to 22.9 (12.0, 53.3) months (*p* < 0.001) was observed. Further, the proportion of cases requiring oxygen supplementation or nasogastric tube placement was lower, and the proportion of cases with suspected bacterial infection was higher in the post-implementation cohort (Table 1). The number of eligible bronchiolitis admissions decreased by 48.1% from pre- to post-implementation (79 vs. 41 admissions). Post-implementation bronchiolitis cases were significantly older (8.4 (5.3, 12.6) vs. 3.8 (2.4, 6.9) months, *p* < 0.001), less frequently required nasogastric tube placement (*p* < 0.001) and had shorter durations of oxygen supplementation (*p* = 0.02). Asthma and pneumonia diagnostic groups were mostly similar in pre- and post-implementation cohorts.

Post-implementation, across all diagnostic groups (Table 2), CLD was associated with significantly longer oxygen supplementation durations than SD (*p* = 0.005). When comparing CLD and SD post-implementation stratified by diagnostic group, bronchiolitis and asthma groups were similar. The CLD pneumonia diagnostic group had lower antibiotic usage rates (*p* = 0.009), but similar rates of suspected bacterial infections, RSV positivity and CRP values. Comparisons of CLD and pooled pre- and post-implementation SD admissions are available as supplemental Table S1.

### Safety outcomes

No deaths or unexpected complications occurred across all admissions. Fourteen-day readmission rates were comparable between CLD (3.2%), pooled SD (3.4%) and post-implementation SD (4.5%) cohorts (Tables 3 and 4, supplemental Table S2). Emergency department (ED) reassessment rates were also comparable, with 4.8% in CLD versus 7.6% in pooled SD and 5.7% in post-implementation SD. Among the CLD cohort, two readmissions occurred, both after bronchiolitis discharges and due to suspected bacterial superinfection. Additionally, one CLD-pneumonia patient had two symptom-related ED reassessments without readmission. In the post-implementation SD cohort, four readmissions occurred: one each after bronchiolitis and viral pneumonia with suspected bacterial superinfection, and two after asthma discharge with symptom recurrence. In addition, one patient was reassessed twice for pleuropneumonia without readmission.

**Table 3:** Outcome table comparing pre- and post-implementation cohorts stratified by diagnostic group

	Overall	All diagnoses		*p*-value	Bronchiolitis		*p*-value	Pneumonia		*p*-value	Asthma/obstructive bronchitis		*p*-value
Number of cases	352	352			120			134			98		
CLD implementation period	All	Pre	Post		Pre	Post		Pre	Post		Pre	Post	
**Number**	**352**	**202**	**150**		**79**	**41**		**67**	**67**		**56**	**42**	
Cases with readmission within 14 days after discharge, *n* (%)	12 (3.4)	6 (3.0)	6 (4.0)	0.82	3 (3.8)	3 (7.3)	0.41	1 (1.5)	1 (1.5)	1	2 (3.6)	2 (4.8)	1
Cases with ED-visits within 14 days after discharge, *n* (%)^a^	25 (7.1)	17 (8.4)	8 (5.3)	0.37	6 (7.6)	3 (7.3)	1	3 (4.5)	3 (4.5)	1	8 (14.3)	2 (4.8)	0.18
Cases with current minor complications/comorbidities, *n* (%)	103 (29.3)	61 (30.2)	42 (28.0)	0.74	16 (20.3)	13 (31.7)	0.24	34 (50.7)	20 (29.9)	0.02	11 (19.6)	9 (21.4)	1
Median time of day of discharge HH:MM [IQR]	12:46 [11:20, 14:37]	12:46 [11.15, 14.00]	13:29 [11:38, 15:00]	0.004	12:22 [11.21, 14.11]	13:10 [11:23, 14:49]	0.47	12:46 [11:38, 14:00]	13.20 [11:45, 14:59]	0.17	12:21 [11:00, 13:32]	13:52 [11:43, 15:26]	0.004
Median length of hospital stay, days [IQR]	2.2 [1.5, 3.8]	2.7 [1.7, 4.4]	1.8 [1.1, 2.8]	< 0.001	3.9 [2.4, 5.5]	2.0 [1.3, 4.3]	0.003	2.7 [1.9, 3.8]	1.8 [1.1, 2.8]	< 0.001	1.8 [1.4, 2.7]	1.7 [1.1, 2.2]	0.13
Median duration of oxygen stop to discharge, hours [IQR]^b^	22.8 [13.7, 28.6]	25.4 [18.4, 29.5]	19.7 [10.1, 27.2]	< 0.001	26.3 [19.5, 36.2]	18.4 [11.0, 25.8]	0.12	25.6 [19.0, 28.1]	20.6 [9.5, 27.6]	0.07	22.8 [16.9, 26.7]	15.1 [9.8, 26.9]	0.005

^a^Including subsequent readmissions

^b^Subanalysis for individuals that needed supplemental oxygen during hospitalisation

**Table 4:** Outcome table of post-implementation cohort, comparing CLD and SD cohorts stratified by diagnostic group

	Overall	All diagnoses		*p*-value	Bronchiolitis		*p*-value	Pneumonia		*p*-value	Obstructive bronchitis/asthma		*p*-value
Number of cases	150	150			41			67			42		
Method of discharge	All	CLD	SD		CLD	SD		CLD	SD		CLD	SD	
**Number**	**150**	**62**	**88**		**22**	**19**		**25**	**42**		**15**	**27**	
Cases with readmission within 14 days after discharge, *n* (%)	6 (4.0)	2 (3.2)	4 (4.5)	1	2 (9.1)	1 (5.3)	1	0 (0.0)	1 (2.4)	1	0 (0.0)	2 (7.4)	0.53
Cases with ED-visits within 14 days after discharge, *n* (%)^a^	8 (5.3)	3 (4.8)	5 (5.7)	1	2 (9.1)	1 (5.3)	1	1 (4.0)	2 (4.8)	1	0 (0.0)	2 (7.4)	0.53
Cases with current minor complications/comorbidities, *n* (%)	42 (28.0)	15 (24.2)	27 (30.7)	0.49	7 (31.8)	6 (31.6)	1	5 (20.0)	15 (35.7)	0.28	3 (20.0)	6 (22.2)	1
Median time of day of discharge, HH:MM [IQR]	13:29 [11:38, 15:00]	13:14 [10:11, 15:34]	13:33 [11:49, 14:58	0.52	13:00 [10:04, 14:53]	13:36 [11:43, 14.40]	0.55	13:02 [10:00, 15:44]	13:25 [12:02, 14:57]	0.40	14:50 [12:22, 15:38]	13:36 [11:35, 15:18]	0.63
Median length of hospital stay, days [IQR]	1.8 [1.1, 2.3]	2.0 [1.5, 3.3]	1.7 [1.1, 2.6]	0.01	2.3 [1.8, 4.9]	1.5 [0.9, 2.7]	0.06	2.0 [1.6. 2.9]	1.7 [1.1, 2.7]	0.37	1.6 [1.3, 2.7]	1.8 [1.0, 2.2]	0.59
Median duration of oxygen stop to discharge, hours [IQR]^b^	19.7 [10.1, 27.2]	15.0 [7.4, 25.2]	21.3 [12.5, 27.6]	0.04	18.1 [8.0, 24.6]	19.6 [11.8, 30.0]	0.38	16.1 [8.6, 26.4]	22.3 [16.4, 27.5]	0.23	9.9 [7.0, 22.6]	22.0 [13.3, 27.6]	0.12

^a^Including subsequent readmissions

^b^Subanalysis for individuals that needed supplemental oxygen during hospitalisation

Rates of minor complications or comorbidities were 24.2% for bronchiolitis, 40.3% for pneumonia and 20.4% for asthma diagnostic groups (supplemental Table S2, Box S7). Rates were similar between the pre- and post-implementation cohorts and CLD and SD, except for the pneumonia diagnostic group, which had lower complication rates post-implementation.

### Patient flow measures

**Fig. 2:**
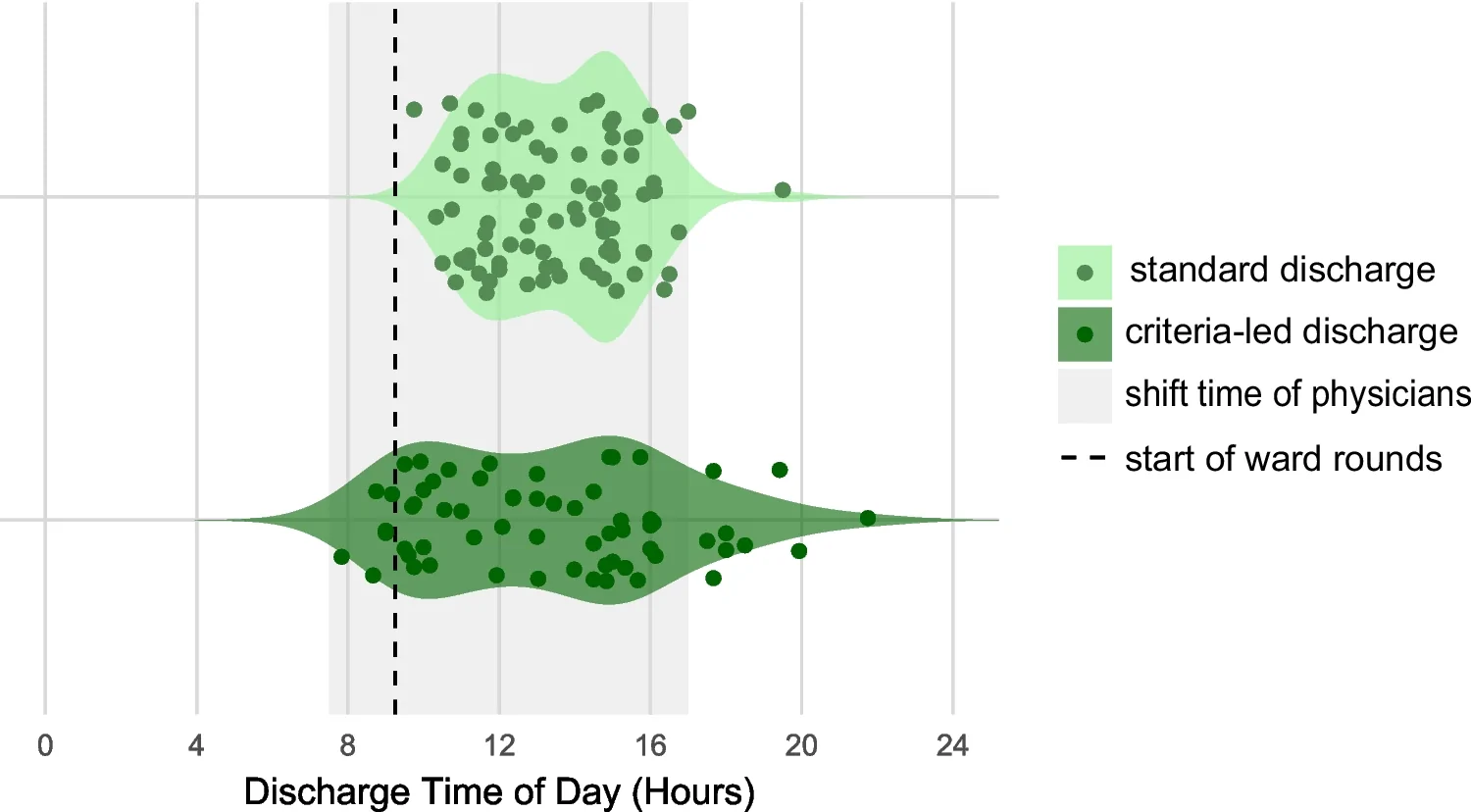
Violin plot with data points showing discharge times of patients with standard discharge compared to criteria-led discharge in the post-implementation period

Post-implementation discharges were completed later than pre-implementation (*p* = 0.004, Table 3). CLD discharge times showed greater variability throughout the day compared to SD post-implementation (Fig. 2), whereas the median time of day of discharge remained similar (Table 4). Discharges before ward round start (9:15 am) rose from 0% in SD to 9.7% (6/62) in CLD (*p* = 0.004).

Length of stay decreased significantly between pre-implementation and post-implementation periods for the diagnostic groups of bronchiolitis and pneumonia (Table 3). Post-implementation, the CLD cohort had a significantly longer median length of stay compared to the SD cohort when analysed across pooled diagnoses (Table 4). Stratified by diagnostic group, no significant differences between CLD and SD were observed. In the multivariable adjusted linear regression model, discharge mode was not associated with length of stay in the post-implementation cohort (coefficient 1.05, 95% CI: 0.89 to 1.23, *p* = 0.58).

In 80% of admissions in the post-implementation period, oxygen supplementation was required. Median time (IQR) from oxygen discontinuation to discharge was 19.7 h (10.1, 27.2) (Table 4). Across pooled diagnostic groups, the CLD cohort had a significantly shorter median time from oxygen discontinuation to discharge (*p* = 0.04). However, this difference was not significant when analysed stratified by diagnostic group. In the multivariable adjusted linear regression model of the post-implementation cohort requiring oxygen supplementation, the discharge mode was not associated with the time to discharge after oxygen discontinuation (coefficient 0.87, 95% CI: 0.68 to 1.12, *p* = 0.29).

### Adherence to discharge criteria

Completion of discharge criteria was similar for CLD and SD post-implementation (supplemental Table S3).

### Patient/caregiver satisfaction

Twenty subjects completed the feedback survey: 18 following CLD (response rate: 26.8%), 1 following SD after non-consent (response rate: 20.0%) and 1 following SD discharge after CLD-discontinuation (response rate: 33.3%). Results are presented in supplemental Table S4. Overall satisfaction was high. All felt safe about going home.

### Staff satisfaction

The baseline survey (December) was answered by 25 employees: 16 nurses and 9 physicians (2 junior and 7 senior). The follow-up survey (March) was answered by 29 employees: 21 nurses and 8 physicians (4 junior and 4 senior). Responses are available in supplemental File 2.

Attitudes toward CLD at baseline were positive. Self-reported workload decreased from baseline to follow-up (*p* < 0.001), with 100% reporting “rather” (60%) to “very high” (40%) workload at baseline versus 83% at follow-up (76% “rather”, 7% “very high”). Most physicians (75%) attributed CLD with reduced workload, while nurses’ responses varied (14% decrease, 57% no change, 29% increase). Stress on routine working days decreased (*p* < 0.001) from 92% reporting stress at baseline to 52% at follow-up. Half of physicians, including 75% of juniors, reported decreased stress due to CLD, whereas nurses’ responses were mixed (19% decrease, 24% increase, 57% no change). Work satisfaction remained similar between timepoints (*p* = 0.18). Seventy-nine percent stated CLD facilitates discharge. At follow-up, nurses were “rather” to “very happy” (71%) with their acquired competencies and felt confident (76%) when executing them. Ninety percent (100% of physicians, 86% of nurses) voted to retain the CLD system.

## Discussion

### Summary

With this study, we confirm safety of CLD in our paediatric inpatient setting. Post-implementation, discharge efficiency improved subjectively, and satisfaction among staff, patients and caregivers was high.

### Interpretation

To our knowledge, we are the first to introduce and evaluate CLD in inpatient paediatrics in Switzerland, and to systematically evaluate staff and patient satisfaction with it.

Following CLD implementation, we observed no increase in ED reassessments, readmissions or complications. As the sole paediatric hospital in our region, we consider our numbers representative and conclude that CLD is safe, consistent with previous reports in other settings [8–11]. This is supported by nurses’ high confidence in managing discharges and families’ strong sense of safety.

We hypothesise that safety may even increase through the structured discharge teaching introduced with CLD, similar to reports of enhanced post-discharge safety following standardised asthma discharge protocols [18].

The degree of CLD standardisation is relevant to safety. We implemented CLD with high standardisation, thereby shaping our target population. As in other studies [2, 19], CLD was limited to straightforward cases and three diagnostic groups. Expanding toward individualisation [20] could broaden eligibility but would likely require advanced nursing roles for implementation.

Contrary to previous paediatric [9, 11] and non-paediatric [21–23] reports, we did not observe shorter length of stay with CLD. Although length of stay decreased significantly from pre- to post-implementation and staff perceived improved efficiency, the CLD cohorts were not discharged earlier than the SD cohorts. Several factors may explain these findings. With CLD, guidance on discharge thresholds was established. Although intended for CLD, these thresholds may also have been applied to SD patients, aligning discharge timing and masking CLD effects. Further, the definition of discharge criteria has been shown to improve efficiency also without delegation of the final discharge decision [19, 24, 25]. CLD may therefore have contributed to the overall reduction in length of stay observed from pre- to post-implementation. However, the introduction of RSV immunisation [14, 26–28] and a lean transformation of the intervention ward may also have shortened the length of stay between periods. The impact of RSV immunisation is reflected in the substantial shift in the bronchiolitis population between periods, limiting the adequacy of pre-implementation bronchiolitis admissions as a comparative cohort with respect to length of stay.

As physicians selected the discharge type, selection bias may further have influenced post-implementation length of stay comparisons. This possibility is supported by longer oxygen supplementation in the diagnosis-pooled CLD cohort compared to SD, despite standardised nurse-led oxygen management for both.

Other efficiency indicators, such as time from oxygen discontinuation to discharge, point to a faster discharge with CLD, although not statistically significant in regression analysis.

Among CLD cohorts, discharge times of day were more variable and detached from ward rounds. Caregiver/patient feedback indicated no disadvantage associated with altered discharge times, and staff even reported improved workload distribution and reduced stress during the ward rounds.

The literature on optimal discharge time of day is contradictory. Fixed morning or discharge before noon (DBN) policies were shown to reduce length of stay [29–31], decrease ED overcrowding [31–33] and benefit patients [34]. However, DBN policies have also been associated with increased length of stay due to metric disincentives [32, 35–37]. For caregivers, postponing discharge to the next day may increase stress by requiring rearrangement of coinciding responsibilities [38]. We believe that immediate discharge upon reaching readiness benefits patients and families while reducing ward-round time pressure.

Patient and caregiver satisfaction with CLD was high. Staff satisfaction was also high and especially junior physicians benefited from the clarified discharge thresholds and shift of workload to nursing staff. While ultimately freeing resources, nurses intermittently faced higher workloads. Despite this imbalance, feedback was positive, and the expanded nursing competencies were broadly welcomed.

### Limitations

Generalisability of our findings is limited by regional characteristics of our healthcare system. Retrospective diagnostic group assignment may have introduced hindsight bias. To minimise this, we applied strict definitions and used physician-documented prioritisation.

The pre-implementation cohort, intended to be used as unbiased primary control group, was affected by population changes following RSV immunisation and a lean transformation coinciding with CLD implementation. While suitable for primary safety comparisons in pneumonia and asthma admissions, it no longer represented an appropriate control for patient flow metrics, which were therefore analysed within the post-implementation cohort, acknowledging potential selection bias.

Voluntary survey participation may have introduced self-selection bias. For staff surveys, interdisciplinary promotion and anonymity aimed to mitigate this and encourage honest feedback.

## Conclusions

CLD optimised and standardised paediatric discharges while maintaining patient safety. Satisfaction and acceptance were high. Although local adaptation is essential, the core concept is broadly transferable. With digitalisation, automated implementation and partial automation of criteria checks could further reduce staff workload and represent a future direction.

## Supplementary Information

Below is the link to the electronic supplementary material.

ESM 1 (PDF 253 KB)

ESM 2 (PDF 0.99 MB)

## Data Availability

Data is available upon reasonable request.

## Abbreviations

CLD: Criteria-led discharge
ED: Emergency department
EHR: Electronic health record
RSV: Respiratory syncytial virus
SD: Standard discharge
SQUIRE: Standards for Quality Improvement Reporting Excellence
