# Supplementary material for: Criteria-led discharge in paediatric inpatient care in Switzerland: a quality improvement study of implementation, safety and satisfaction
Source: Eur J Pediatr. 2026 Nov 7;185(12):921. doi: 10.1007/s00431-026-07578-y (PMC13744470; doi:10.1007/s00431-026-07578-y)
Supplement: Supplementary file 1 [file 431_2026_7578_MOESM1_ESM.pdf]

Criteria-Led Discharge in Paediatric Inpatient Care in Switzerland: A Quality Improvement Study of Implementation, Safety and Satisfaction

SUPPLEMENTAL FILE 2

Submitted to: European Journal of Pediatrics

Authors: Mirjam Kissling[1,2] (submitting author, Mirjam.kissling@luks.ch, ORCID: 0000-0002-2403-7954), Anna Schoeni[1,3] (ORCID: 0000-0002-2421-5925), Sibylle Jung[3], Karin Roth[3], Birgit Wernz[3], Marco Lurà[3], Nicole Ritz[3,4] (corresponding author, ORCID: 0000-0002-1498-1685, Nicole.ritz@unibas.ch)

1 Children's Hospital of Central Switzerland, Center for Child Health Analytics (CCHA), Lucerne, Switzerland
2 University of Lucerne, Faculty of Health Sciences and Medicine, Lucerne, Switzerland
3 Children's Hospital of Central Switzerland, Department of Paediatrics, Lucerne, Switzerland
4 University Children's Hospital Basel and Department of Clinical Research University of Basel, Mycobacterial and Migrant Health Research Group, Basel, Switzerland

QUESTIONNAIRES AMONGST PERSONNEL

Questions and count of answers (n), translated into English from German

Timepoints when questions were asked	Profession
**Both questionnaires: Baseline & FU**<br>**Only baseline (BL)**<br>**Only follow-up (FU)**	Junior physician<br>Senior physician<br>Healthcare assistant<br>Certified nurse

1. What is your profession? (German: Zu welcher Berufsgruppe gehörst Du?)

Response possibilities	answers BL	answers FU
a. Junior doctor	2	4
b. Senior doctor	7	4
c. Medical student	0	0
d. Healthcare assistant	1	0
e. Certified nurse	15	21
f. Nursing student	0	0

Section 1: Stress, workload, satisfaction with working day

2. How happy are you with the structure of your current working day? (German: Wie zufrieden bist Du mit dem aktuellen Ablauf Deines Arbeitstages?)

Response possibilities	Answers BL	Answers FU
a. Very unhappy	1 (0/0 /0 /1)	0 (0/0/0/0)
b. Rather unhappy	7 (0/0 /0 /7)	6 (0/0/0/0)
c. Neither nor	6 (1/1 /0 /4)	4 (0/0/0/0)
d. Rather happy	10 (1/5 /1 /3)	19 (4/2/13/0)
e. Very happy	1 (0/1 /0 /0)	0 (0/0/0/0)

3. Did your satisfaction with the structure of your working day improve or worsen with the introduction of CLD? (German: Hat sich Deine Zufriedenheit mit deinem Arbeitstag durch KIBA verschlechtert / verbessert?)

Response possibilities		Answers FU
a. Clearly worsened		0 (0/0/0/0)
b. Slightly worsened		0 (0/0/0/0)
c. Neither nor		14 (1/2/0/11)
d. Slightly improved		12 (2/0/0/8)
e. Clearly improved		3 (1/2/0/2)

4. How is your general workload? (German: Wie beurteilst Du Deine generelle Arbeitsbelastung?)

Response possibilities	Answers BL	Answers FU
a. Very low	0 (0/0/0/0)	0 (0/0/0/0)
b. Rather low	0 (0/0/0/0)	0 (0/0/0/0)
c. Neutral	0 (0/0/0/0)	5 (0/1/0/4)
d. Rather high	15 (2/4/1/8)	22 (3/3/0/16)
e. Very high	10 (0/3/0/7)	2 (1/0/0/1)

5. Did your workload increase / decrease with CLD introduction? (German: hat sich Deine Arbeitsbelastung durch KIBA erhöht / erniedrigt?)

Response possibilities		Answers FU
a. Strongly decreased		2 (1/0/0/1)
b. Slightly decreased		7 (3/2/0/2)
c. Similar to pre-implementation		14 (0/2/0/12)
d. Slightly increased		5 (0/0/0/5)

e. Significantly increased		1 (0/0/0/1)

6. How do you assess your stress level on a typical working day? (German: Wie beurteilst Du das Stresslevel Deines typischen Arbeitstages?)

Response possibilities	Answers BL	Answers FU
a. Very relaxed	0 (0/0/0/0)	0 (0/0/0/0)
b. Rather relaxed	0 (0/0/0/0)	1 (0/1/0/0)
c. Neutral	2 (0/1/0/1)	13 (1/2/0/10)
d. Rather stressed	21 (2/6/1/12)	14 (3/1/0/10)
e. Very stressed	2 (0/0/0/2)	1 (0/0/0/0)

7. Did the introduction of CLD change your stress level? (German: Hat die Einführung von KIBA dein Stresslevel verändert?)

Response possibilities		Answers FU
a. Significantly increased		1 (0/0/0/1)
b. Slightly increased		4 (0/0/0/4)
c. Neutral		16 (1/3/0/12)
d. Slightly decreased		6 (2/1/0/3)
e. Significantly decreased		2 (1/0/0/1)

Section 2: collaboration and communication concerning discharges

8. How good/bad is the communication / collaboration between doctors and nurses concerning discharges at the moment? (German: Wie gut / schlecht funktioniert die Kommunikation / Zusammenarbeit zwischen ÄrztInnen und Pflegenden im Hinblick auf Austritte zum jetzigen Zeitpunkt?)

Response possibilities	Answers BL	Answers FU
a. Very bad	0 (0/0/0/0)	0 (0/0/0/0)
b. Rather bad	9 (0/0/1/8)	7 (1/1/0/5)
c. Neutral	6 (0/1/0/5)	13 (2/0/0/11)
d. Rather good	9 (1/6/0/2)	8 (0/3/0/5)
e. Very good	1 (01/0/0/0)	1 (1/0/0/0)

9. In what areas does communication work well concerning discharges?
   a. Free-text option
10. In what areas is communication / collaboration insufficiently working concerning discharges?
   a. Free-text option
11. What measures could improve communication / collaboration?
   a. Free-text option
12. Did the introduction of CLD change the quality of interprofessional communication? (Hat die Einführung von KIBA eine Verbesserung / Verschlechterung bezüglich der interdisziplinären Kommunikation bewirkt?)

Response possibilities		Answers FU
a. Clearly worsened with CLD		0 (0/0/0/0)
b. Slightly worsened with CLD		0 (0/0/0/0)
c. Stayed similar		18 (3/3/0/12)
d. Slightly improved with CLD		8 (1/0/0/7)
e. Clearly improved with CLD		3 (0/1/0/2)

Section 3: General discharge management

13. How efficient do you think our discharges are in general? (starting at recognition of discharge readiness, to organization of discharge, until discharge completion) (German: Wie effizient läuft Deiner Meinung nach der Austritt unserer Patienten? (Von der Erfassung der Austrittsfähigkeit, über die Organisation des Austrittes bis zur effektiven Entlassung.))

Response possibilities	Answers BL	Answers FU
a. Very inefficient	0 (0/0/0/0)	0 (0/0/0/0)
b. Rather inefficient	9 (0/3/0/6)	19 (1/2/0/16)
c. Neither inefficient nor efficient	9 (0/1/1/7)	4 (0/1/0/3)

d.	Rather efficient	7 (2/3/0/2)	6 (3/1/0/2)
e.	Very efficient	0 (0/0/0/0)	0 (0/0/0/0)

14. What are the main reasons for discharge delay in "discharge ready" patients? Please choose the most important reasons (maximum 3). (German: Was sind die wichtigsten Gründe für Verzögerungen eines Austritts bei "austrittsfähigen" PatientInnen? Wähle bitte die Deiner Meinung nach relevantesten Gründe an (max. 3).)

Response possibilities		Answers BL	Answers FU
a.	Delay due to missing documents (e.g. prescriptions)	15	14
b.	Delay due to social aspects (e.g. lack of childcare options, lack of support at home, challenging social circumstances, parent's concern for safety/external support)	5	7
c.	Delay due to missing transport	5	6
d.	Delay due to wait for ward rounds	22	20
e.	Delay due to missing capacities for procedures (e.g. dressing changes, removal of lines, suture removal),	2	4
f.	Delay due to missing consults	15	18
g.	Delay due to organizational issues of follow-up with local physicians (appointment at local pediatrician)	0	0
h.	Delay due to difficulties with education/instruction/communication	7	4
i.	Other: free-text option	• Necessary discussion between junior and senior physician after ward rounds (1)<br>• Delay due to missing resources of junior physician	• Necessary discussion between junior and senior physician after ward rounds (3)<br>• Delay due to missing lab results (1)

15. How certain are you, that the timepoint of discharge of your patients is well chosen? (German: Wie sicher bist Du Dir bei der Entlassung Deiner PatientInnen, dass der Zeitpunkt der Entlassung richtig gewählt ist?)

Response possibilities		Answers BL	Answers FU
a.	Very uncertain	0 (0/0/0/0)	0 (0/0/0/0)
b.	Rather uncertain	0 (0/0/0/0)	0 (0/0/0/0)
c.	Neither certain nor uncertain	6 (0/0/1/5)	1 (0/0/0/01)
d.	Rather certain	13 (2/1/0/10)	23 (3/3/0/17)
e.	Very certain	6 (0/6/0/0)	5 (1/1/0/3)

Introductory text for the next section (questions 16-18): The following questions refer to discharges of children who qualify for a criteria-led discharge. This concerns patients without pre-existing conditions, without complications in the current disease course, who are hospitalised due to:

- **obstructive bronchitis,**
- **asthma,**
- **bronchiolitis,**
- **pneumonia,**
- **urinary tract infection,**
- **febrile seizure,**
- **caffeine withdrawal,**
- **vaccinations in the context of brady-apnea syndrome, or**
- **gastroenteritis.**

Remark: We received oral feedback from several employees on questions 16-18, that they did not understand them properly. We thus did not include questions 16 – 18 into analysis for this study.

16. How efficient do you think our discharges are for CLD patients? (starting at recognition of discharge readiness, to organization of discharge until discharge completion) (German: Wie effizient läuft Deiner Meinung nach das aktuelle Austrittsmanagement bei KIBA-Patienten? (Von der Erfassung der Austrittsfähigkeit über die Organisation des Austrittes bis zur effektiven Entlassung.)

**Response possibilities**	**Answers BL**	
a. Very inefficient	0 (0/0/0/0)	
b. Rather inefficient	8 (0/1/0/7)	
c. Neither inefficient nor efficient	8 (0/3/1/4)	
d. Rather efficient	6 (0/2/0/4)	
e. Very efficient	3 (2/1/0/0)	

17. What are the main reasons for discharge delay in "discharge ready" CLD patients? Please choose the most important reasons (maximum 3). (German: Wie effizient läuft Deiner Meinung nach das aktuelle Austrittsmanagement bei KIBA-Patienten? (Von der Erfassung der Austrittsfähigkeit über die Organisation des Austrittes bis zur effektiven Entlassung.) Wähle bitte die Deiner Meinung nach relevantesten Gründe an (max. 3).)

**Response possibilities**	**Answers BL**	
a. Delay due to missing documents (e.g. prescriptions)	12	
b. Delay due to social aspects (e.g. lack of childcare options, lack of support at home, challenging social circumstances, parent's concern for safety/external support)	4	
c. Delay due to missing transport	6	
d. Delay due to wait for ward rounds	7	
e. Delay due to missing capacities for procedures (e.g. dressing changes,	1	

removal of lines, suture removal),		
f. Delay due to missing consults	5	
g. Delay due to organizational issues of follow-up with local physicians (appointment at local pediatrician)	0	
h. Delay due to difficulties with education/instruction/communication	6	
i. Other: free-text option	• Missing physician resources (1)<br>• No judgement possible (4)<br>• Insecurities of nurses (2)<br>• Only rarely delays (1)<br>• Wait for CLD decision (1)	

18. How certain are you, that the timepoint of discharge of your CLD-patients is well chosen? (Wie sicher bist Du Dir bei der Entlassung Deiner KIBA-PatientInnen, dass der Zeitpunkt der Entlassung richtig gewählt ist?)

**Response possibilities**	**Answers BL**	
a. Very uncertain	0 (0/0/0/0)	
b. Rather uncertain	1 (0/0/0/1)	
c. Neither certain nor uncertain	7 (0/1/1/5)	
d. Rather certain	12 (1/2/0/9)	
e. Very certain	5 (1/4/0/0)	

19. What attitude do you have towards the CLD-project? (German: Wie ist Deine Einstellung gegenüber dem KIBA-Projekt?)

**Response possibilities**	**Answers BL**	
a. Negative / critical	0	
b. Rather negative / critical	2 (0/0/0/2)	
c. Neutral	1 (0/0/0/1)	
d. Rather positive / approving	14 (2/3/1/8)	
e. Positive / approving	8 (0/4/0/4)	

20. What are your main concerns with CLD? (German: Was sind Deine grössten Bedenken bezüglich KIBA?)
    a. Free-text option
21. What kind of teaching / information are you still missing on CLD? (German: Welche Information / Teaching fehlt Dir bisher noch zum KIBA?)
    a. Free-text option

22. Does CLD facilitate discharge management for your patients in your view? (German: Erleichtert KIBA das Austrittsmanagement Deiner PatientInnen aus Deiner Sicht?)

Response possibilities		Answers FU
a. Not at all		0 (0/0/0/0)
b. Rather not		0 (0/0/0/0)
c. Neutral		6 (0/1/0/5)
d. Rather yes		17 (3/3/0/11)
e. Very much so		6 (1/0/0/5)

23. If you could decide: would you keep CLD as a system or get rid of it again? (German: Wenn Du entscheiden könntest: Würdest Du KIBA beibehalten oder wieder abschaffen?)

Response possibilities		Answers FU
a. Keep		26 (4/4/0/18)
b. Get rid of it		3 (0/0/0/3)

24. Comments: Free-text option

Section 3: Organizational topics

25. How happy are you with available teachings on CLD? Grade from 1-5

Response possibilities		Answers FU
a. 1		0 (0/0/0/0)
b. 2		0 (0/0/0/0)
c. 3		5 (1/1/0/3)
d. 4		11 (0/3/0/8)
e. 5		13 (3/0/0/10)

26. What information / teaching are you missing on CLD? What additional wishes on teaching or execution of CLD do you wish for?
    a. Free-text Option

Section 4: feedback from nursing staff: *The following questions were only answered by nurses*

27. How happy are you with the expansion of competencies for nurses with CLD? (Für Pflegende: Wie zufrieden bist Du mit der Kompetenzerweiterung in der Pflege durch KIBA?)

Response possibilities		Answers FU
a. Very unhappy		0
b. Rather unhappy		1
c. Neutral		5
d. Rather happy		5
e. Very happy		10

28. Do you feel confident / secure when using these expanded nursing skills? (German: Fühlst Du Dich sicher im Umgang mit den erweiterten Kompetenzen in der Pflege?)

Response possibilities		Answers FU
a. Very insecure		0
b. Rather insecure		3
c. Neutral		2
d. Rather secure		10
e. Very secure		6

29. What would help increase your confidence / security?
    a. Free-text option
