# Supplementary material for: Criteria-led discharge in paediatric inpatient care in Switzerland: a quality improvement study of implementation, safety and satisfaction
Source: Eur J Pediatr. 2026 Nov 7;185(12):921. doi: 10.1007/s00431-026-07578-y (PMC13744470; doi:10.1007/s00431-026-07578-y)
Supplement: Supplementary file 2 [file 431_2026_7578_MOESM2_ESM.pdf]

Criteria-Led Discharge in Paediatric Inpatient Care in Switzerland: A Quality Improvement Study of Implementation, Safety and Satisfaction

SUPPLEMENTAL FILE 1

Submitted to: European Journal of Pediatrics

Authors: Mirjam Kissling[1,2] (submitting author, Mirjam.kissling@luks.ch, ORCID: 0000-0002-2403-7954), Anna Schoeni[1,3] (ORCID: 0000-0002-2421-5925), Sibylle Jung[3], Karin Roth[3], Birgit Wernz[3], Marco Lurà[3], Nicole Ritz[3,4] (corresponding author, ORCID: 0000-0002-1498-1685, Nicole.ritz@unibas.ch)

1 Children's Hospital of Central Switzerland, Center for Child Health Analytics (CCHA), Lucerne, Switzerland
2 University of Lucerne, Faculty of Health Sciences and Medicine, Lucerne, Switzerland
3 Children's Hospital of Central Switzerland, Department of Paediatrics, Lucerne, Switzerland
4 University Children's Hospital Basel and Department of Clinical Research University of Basel, Mycobacterial and Migrant Health Research Group, Basel, Switzerland

Supplemental File 1

Table of contents

Box S1 Discharge criteria by diagnostic group 3
Box S2 List of variables / data points extracted from EHR 4
Box S3 Criteria for assignment of diagnostic groups 5
Box S4 list of analysed traceable discharge criteria and methods of analysis 6
Box S5 Covariates, with respective transformations applied if applicable, used for adjustment of length of stay model 7
Box S6 Covariates, with respective transformation applied if applicable, used for adjustment of model assessing time to discharge after supplemental oxygen discontinuation in patients with any oxygen supplementation during hospitalisation 8
Box S7 Detailed list (count) of minor comorbidities or complications by diagnostic group; some patients may have had several of the listed 9
Box S8 Result of nasopharyngeal swabs by diagnostic group for all implementation periods 10

Fig. S1 Participants included pre- and post-implementation of criteria-led discharge with reasons for exclusion 11
Fig. S2 Participants included pre- and post-implementation of criteria-led discharge, with list of current and chronic comorbidities / complications leading to exclusion 12

Table S1 Baseline characteristics stratified by diagnostic group and discharge method; SD includes both pre- and post-implementation cohort 13
Table S2 Outcome table comparing CLD and SD cohorts, stratified by diagnostic group; SD includes both pre- and post-implementation cohort 14
Table S3: Fulfilment of disease specific discharge criteria, comparing post-implementation CLD and SD cohorts at discharge 15
Table S4: Survey results of caregiver / patient satisfaction with discharge 16

1.1. Boxes

Discharge Criteria		
**For all diagnostic groups**		
- Good general condition<br>- No supplemental oxygen and oxygen-saturations ≥ 90% on room air for at least 6 hours<br>- Last medical chart review less than 24h ago and last clinical bedside review less than 48 hours ago<br>- Organized aftercare at local paediatrician within 48h after discharge<br>- Any additional criteria defined by physician at prescription fulfilled		
**Bronchiolitis**	**Pneumonia**	**Obstructive bronchitis / asthma**
- No signs of respiratory distress (tachypnoea, retractions) at rest<br>- Drinking quantity: at least 2/3 of usual drinking quantity for the last 12 hours reached	- Normal drinking quantity (like at home)<br>- If antibiotic course is necessary: able to take and well tolerated oral therapy<br>- Body temperature in last 24h constantly ≤ 38°C.	- No signs of respiratory distress (tachypnoea, retractions) at rest, slight wheezing is accepted<br>- Normal drinking quantity (like at home)<br>- Inhalation interval stretched to 4hourly or more with 2-4 puffs max. per inhalation

Box S1 Discharge criteria by diagnostic group

Baseline patient characteristics	• age<br>• sex<br>• weight<br>• gestational age at birth (if available)<br>• height
Diagnoses	• diagnosis marked as primary diagnosis<br>• all active diagnoses (including registered chronic disease)
Admission and discharge information	• admission date and time<br>• discharge date and time<br>• discharge type<br>o CLD-status (accepted, rejected, discontinued; any linked notes to CLD status)<br>o reasons for discontinuing the CLD pathway (if commented on)<br>o start / prescription of CLD in EHR including timestamp<br>• department of admission and departments throughout stay: ED, in-patient ward(s) and PICU
Diagnostics	• result of swabs (PCR or antigen tests):<br>o geneXpert: RSV, Influenza A & B, covid19<br>o multiplex PCR respiratory panel<br>o antigen tests<br>• order of imaging of chest including Xray, sonography and CT<br>• result note / assessment of imaging performed of chest including Xray, sonography and CT.<br>• lab work: complete blood counts and CRP
Respiratory assessment and supportive therapy	• duration of oxygen supplementation and type of application (e.g. nasal canula or reservoir mask)<br>• need for high flow therapy / intubation / cpap<br>• respiration assessment for each day of hospitalisation<br>o retractions + localization of retractions<br>o flaring of the nostrils<br>o tachypnoea<br>o cyanosis (skin assessment)<br>o grunting<br>• oxygen saturations with date and time
Nutrition assessment and supportive therapy	• oral intake: drinking quantity in 24h and 12h before discharge<br>• need for enteral or parenteral nourishment<br>o gastric tube placement<br>o application of fluids enteral or parenteral documented
Further assessments	• body temperature measurements
Medication administration	• cumulative doses of antipyretics per registered weight<br>• antibiotics administered<br>• number and dosages of inhalations necessary throughout hospitalisation

Box S2 List of variables / data points extracted from EHR

<table>
<tr><th>Bronchiolitis definition</th><th>Pneumonia definition</th><th>Asthma / obstructive bronchitis definition</th></tr>
<tr>
<td>a. diagnosis of “bronchiolitis” available for children <2 years of age,
b. patients with diagnosis of “respiratory tract infection” with supplemental oxygen need or respiratory distress AND RSV-positivity in children <2 years of age</td>
<td>a. diagnosis of “pneumonia” available,
b. patients with diagnosis of “respiratory tract infection” with radiological evidence suggesting pneumonia,
c. patients with diagnosis of “respiratory tract infection” with antibiotic treatment without other reason for antibiotics in the list of diagnoses
d. patients with diagnosis of “respiratory tract infection” with need of oxygen supplementation or respiratory distress AND without RSV-proof or bronchodilator treatment.
e. patients ≥2 years of age with “respiratory tract infection” and supplemental oxygen need/respiratory distress and RSV infection
f. patients ≥2 years of age with documented diagnosis of “bronchiolitis”
e) and f) were analysed as viral pneumonia in accordance with international guidelines, where an upper age limit of 2 years for bronchiolitis diagnosis is suggested [1; 2]</td>
<td>a. diagnosis of “obstructive bronchitis”, “acute wheeze” or “asthma” available
b. patients with need of repetitive inhalation due to respiratory distress without fulfilling the definitions of pneumonia or bronchiolitis.

Clarification: Patients meeting criteria for pneumonia or bronchiolitis with additional obstructive symptoms according to the diagnosis list were analysed in their primary subgroup and obstruction was recorded as minor complication of the primary disease.</td>
</tr>
<tr><td colspan="3">- When cohort assignment was ambiguous, the primary (text) diagnosis as recorded by the treating physician served as the key determinant in classification.
- RSV = respiratory syncytial virus</td></tr>
</table>

Box S3 Criteria for assignment of diagnostic groups

List of traceable discharge criteria (*diagnostic groups with applicability*)
1) Sustained oxygen saturations of ≥ 90% for at least 6 hours+,° (*all*),
2) No supplemental oxygen requirement for at least 6 hours+ (*all*),
3) Inhalation intervals of a minimum of 4 hourly+ *(asthma diagnostic group*).
Definition: interval between the last two inhalations and the last inhalation to discharge were considered.
4) Patient afebrile (≤38.0°) for at least 24 hours+ *(pneumonia diagnostic group*).
Definition: interval between last documented fever and discharge.
5) No respiratory distress at rest *(asthma and bronchiolitis diagnostic groups*)
Definition: approximated using age-adjusted tachypnoea thresholds[3; 4].

+ A 10% margin of error was applied to time thresholds, accounting for imprecise documentation.
° Intermittent desaturations of < 30 seconds occurring over less than half an hour were considered normal [2; 5; 6].

Details of analysis:

- All patients were analysed adhering to the selected CLD discharge criteria, even if the retrospectively assigned diagnostic group with the corresponding criteria catalogue differed (e.g. bronchiolitis criteria prescribed, final diagnostic group being pneumonia due to age >2 years).

Box S4 list of analysed traceable discharge criteria and methods of analysis

Demographic covariates	• Age*, ln transformed*<br>• Sex<br>• Known prematurity
Clinical covariates	• Diagnostic group<br>• Suspected bacterial aetiology<br>• Respiratory syncytial virus (RSV) positivity<br>• Minor comorbidity / complication present
Treatment-related covariates	• Need for intensive care unit treatment<br>• Nasogastric tube placement in hospitalisation<br>• Timespan between first and last oxygen supplementation*, ln (x+1) transformed*<br>• Receival of antibiotic treatment<br>• Receival of systemic corticosteroid treatment

Box S5 Covariates, with respective *transformations* applied if applicable, used for adjustment of length of stay model

Demographic covariates	• Age, *ln transformed*<br>• Sex<br>• Known prematurity
Clinical covariates	• Diagnostic group
Treatment-related covariates	• Need for intensive care unit treatment<br>• Nasogastric tube placement in hospitalisation<br>• Tachypnoea at timepoint of oxygen discontinuation (for asthma and bronchiolitis group)<br>• Fulfilment of fever criterion at timepoint of oxygen discontinuation (for pneumonia diagnostic group)<br>• Receival of antibiotic treatment<br>• Timespan between first and last oxygen supplementation, *ln transformed*

Box S6 Covariates, with respective *transformation* applied if applicable, used for adjustment of model assessing time to discharge after supplemental oxygen discontinuation in patients with any oxygen supplementation during hospitalisation

Bronchiolitis	Pneumonia	Asthma / obstructive bronchitis
- obstructive symptoms (12)<br>- otitis media acuta (OMA) (14)<br>- varicella infection (1)<br>- apnoea episodes (1)<br>- conjunctivitis (1)<br>- rash (1)<br>- paronychia (1)<br>- virus associated vomiting (1)<br>- apnoea (1)	- obstructive symptoms (30)<br>- otitis media acuta (OMA) (19)<br>- conjunctivitis (4)<br>- gastroenteritis (2)<br>- evidence of small effusions (3)<br>- acidosis with dehydration (1)<br>- dehydration (1)<br>- rash (1)<br>- impetigo contagiosa (1)<br>- iron deficiency anemia (1)<br>- mild hyponatremia (1)<br>- otitis media acuta perforata (1)<br>- salbutamol-associated lactic acidosis (1)<br>- simple febrile seizure (1)<br>- vulvovaginitis (1)	- Otitis media acuta (13)<br>- Otitis media acuta perforata (1)<br>- Conjunctivitis (1)<br>- Hyperglycaemia (1)<br>- Norovirus coinfection (1)<br>- Tinea corporis (1)<br>- Macrocephaly (1)<br>- Blood pressure hypertension (1)<br>- Viral hepatitis and splenomegaly (1)<br>- Simple febrile seizure (1)

Box S7 Detailed list (count) of minor comorbidities or complications by diagnostic group; some patients may have had several of the listed

**Respiratory pathogens found in bronchiolitis**	**Respiratory pathogens found in pneumonia**	**Respiratory pathogens found in asthma/obstructive bronchitis**
In alphabetical order		
- Influenza infection (1)<br>- RSV (95)<br>- RSV coinfections with<br>o Influenza (1)<br>o Rhino-enterovirus (1)<br>o Covid19 (1)<br><br>None (21)	Exclusively viral agents<br>- hMPV (3)<br>- influenza (13)<br>- parainfluenza (1),<br>- RSV (47),<br>- RSV coinfections with<br>o adenovirus (1),<br>o Covid19 (2)<br>o Influenza (3)<br><br>bacterial and/or mixed agents<br>- chlamydia pneumonia (1)<br>- chlamydia pneumonia coinfections with<br>o Influenza (1)<br>- Mycoplasma pneumoniae (17),<br>- Mycoplasma coinfections with<br>o hMPV and rhino-enterovirus (1),<br>o RSV (1)<br>- Streptococci group A (1)<br>- Streptococcus pneumoniae coinfection with<br>o RSV (1)<br>None (41)	- Influenza (1)<br>- RSV (24)<br>- RSV coinfections with<br>o Influenza (1)<br>- Rhino-/enterovirus (2)<br><br>None (70)

Box S8 Result of nasopharyngeal swabs by diagnostic group for all implementation periods

1.2. Figures

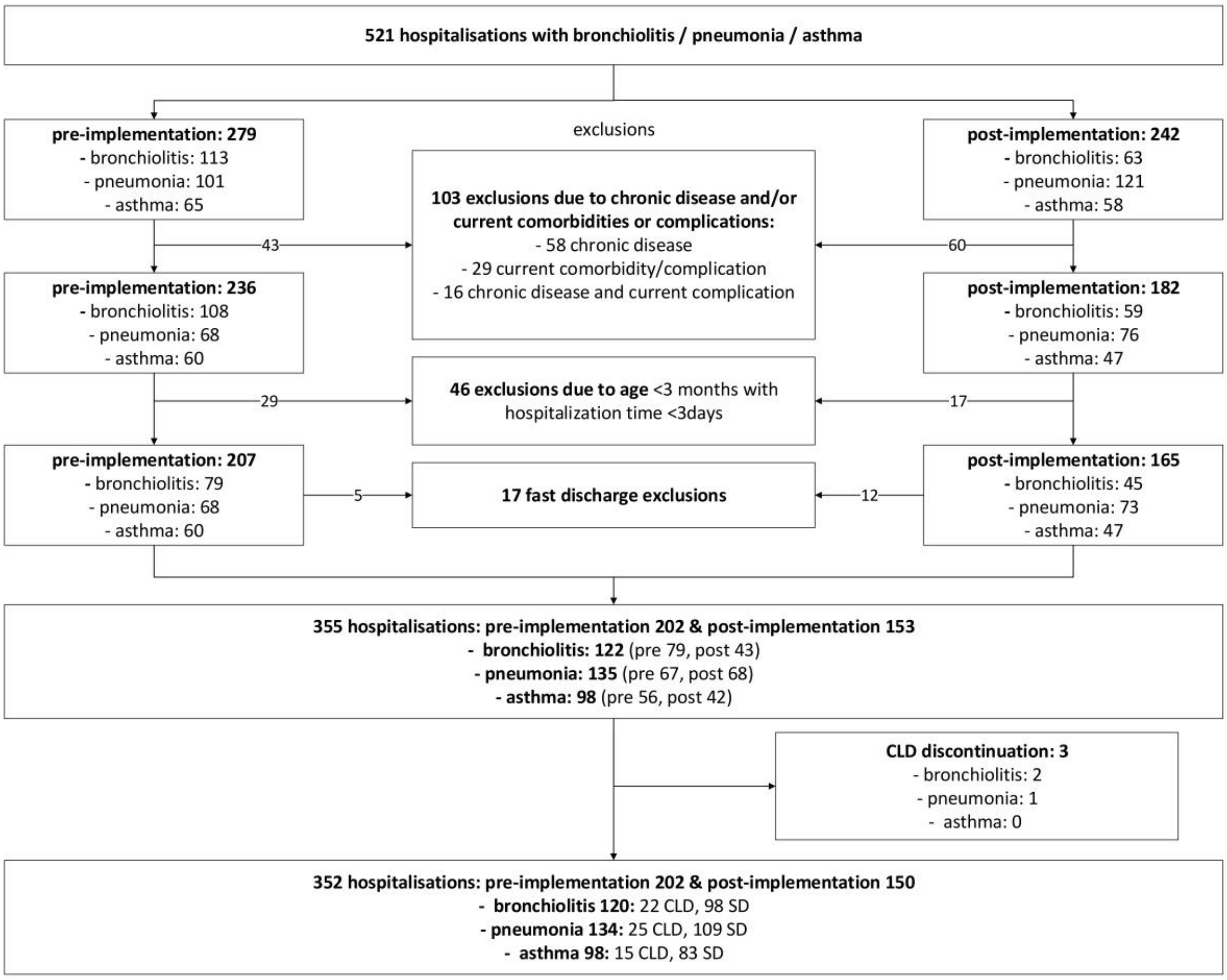

Fig. S1 Participants included pre- and post-implementation of criteria-led discharge with reasons for exclusion

This figure was created using Microsoft Visio.

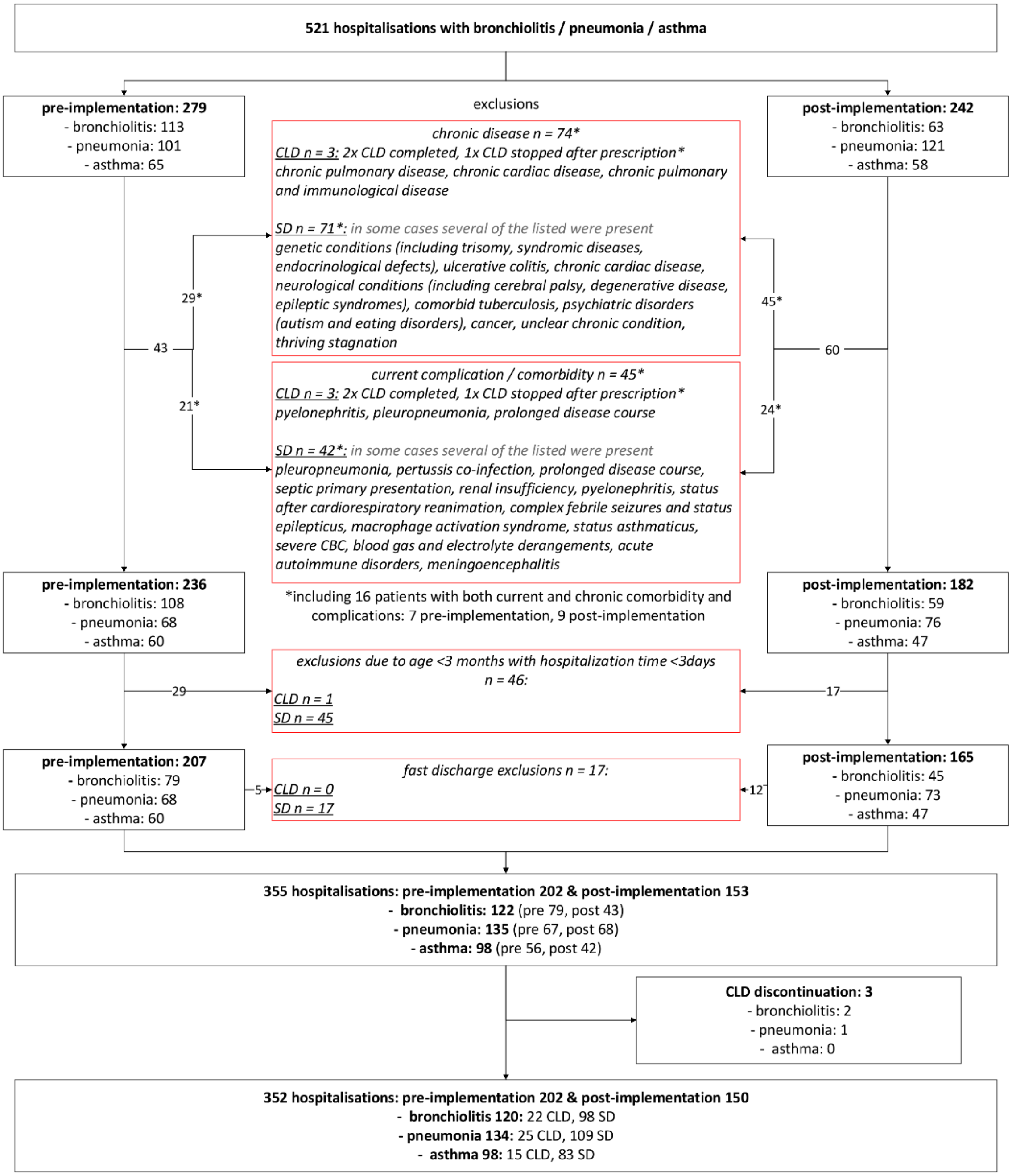

Fig. S2 Participants included pre- and post-implementation of criteria-led discharge, with list of current and chronic comorbidities / complications leading to exclusion

This figure was created using Microsoft Visio.

1.3. Tables

	Overall	All diagnoses		p-value	Bronchiolitis		p-value	Pneumonia		p-value	Obstructive Bronchitis / Asthma		p-value
**Number of cases**	352	352			120			134			98		
**Method of discharge**	**All**	**CLD**	**SD**		**CLD**	**SD**		**CLD**	**SD**		**CLD**	**SD**	
**Number**	**352**	**62**	**290**		**22**	**98**		**25**	**109**		**15**	**83**	
Cohort; pre-implementation, n (%)	202 (57.4)	0 (0)	202 (69.7)		0 (0)	79 (80.6)		0 (0)	67 (61.5)		0 (0)	56 (67.5)	
Female, n (%)	168 (47.7)	35 (56.5)	133 (45.9)	0.17	12 (54.5)	43 (43.9)	0.50	14 (56.0)	51 (46.8)	0.54	9 (60.0)	39 (47.0)	0.52
Age in months, median [IQR]	18.1 [6.9, 44.5]	22.8 [10.4, 55.6]	16.8 [6.4, 42.1]	0.12	7.5 [5.6, 11.6]	4.3 [2.5, 8.5]	0.004	34.4 [20.1, 70.0]	38.7 [16.9, 63.6]	0.38	39.5 [24.9, 63.2]	31.8 [16.8, 52.3]	0.38
Cases with known prematurity, n (%)	40 (11.4)	8 (12.9)	32 (11.0)	0.84	4 (18.2)	11 (11.2)	0.47	1 (4.0)	8 (7.3)	1	3 (20.0)	13 (15.7)	0.70
ICU stay, n (%)	22 (6.2)	4 (6.5)	18 (6.2)	0.25	3 (13.6)	8 (8.2)	0.42	1 (4.0)	6 (5.5)	1	0 (0.0)	4 (4.8)	1
Nasogastric tube placed, n (%)	79 (22.4)	9 (14.5)	70 (24.1)	0.14	6 (27.3)	54 (55.1)	0.03	2 (8.0)	12 (11.0)	1	1 (6.7)	4 (4.8)	0.57
Cases with need of supplemental oxygen during hospitalisation, n (%)	302 (85.8)	53 (85.5)	249 (85.9)	1	18 (81.8)	86 (87.8)	0.49	22 (88.0)	87 (79.8)	0.41	13 (86.7)	76 (91.6)	0.62
Time interval of first to last oxygen supplementation in hours, median [IQR][a]	33.8 [13.6, 66.1]	33.9 [16.1, 59.8]	33.7 [13.1, 68.7]	0.90	28.6 [8.9, 40.7]	57.1 [13.9, 92.5]	0.76	35.9 [14.4, 49.0]	39.2 [16.1, 65.8]	0.56	28.6 [8.9, 40.7]	24.8 [9.5, 36.9]	0.40
Swab results positive for any significant respiratory pathogen, n (%)[b]	220 (62.5)	45 (72.6)	175 (60.3)	0.10	18 (81.8)	81 (82.7)	1	20 (80.0)	73 (67.0)	0.30	7 (46.7)	21 (25.3)	0.17
RSV positivity, n (%)	178 (50.6)	33 (53.2)	145 (50.0)	0.75	17 (77.3)	81 (82.7)	0.78	10 (40.0)	45 (41.3)	1	6 (40.0)	19 (22.9)	0.28
Suspected bacterial infection, n (%)	75 (56.0)	12 (19.4)	63 (21.7)	0.81	0 (0.0)	0 (0.0)	NA	12 (48.0)	63 (57.8)	0.51	0 (0.0)	0 (0.0)	NA
CRP measured, n (%)	170 (48.3)	32 (53.3)	138 (47.6)	0.66	4 (18.2)	39 (43.8)	0.08	20 (80.0)	76 (69.7)	0.43	8 (53.3)	23 (27.7)	0.10
Highest CRP: median [IQR]	28.0 [9.0, 74.0]	32.0 [19.0, 80.8]	24.9 [8.0, 70.8]	0.19	24.0 [18.3, 51.5]	9.5 [6.0, 24.0]	0.09	45.0 [19.3, 94.5]	59.0 [23.2, 111.3]	0.37	25.0 [19.0, 57.0]	11.0 [5.4, 20.0]	0.01
Cases that received resp. antibiotics during hospitalisation, n (%)	98 (27.8)	14 (22.6)	84 (29.0)	0.39	2 (9.1)	5 (5.1)	0.61	11 (44.0)	75 (68.8)	0.04	1 (6.7)	4 (4.8)	0.57
Number of Salbutamol inhalations per hospital day median [IQR]; mean (SD)	1.3 [0.0, 6.2]	1.3 [0.0, 5.8]	1.3 [0.0, 6.2]	0.98	0.7 [0.0, 1.9]	0.0 [0.0, 1.0]	0.03	0.7 [0.0, 1.4]	0.5 [0.0, 3.0]	0.32	7.9 (2.6)	7.4 (2.2)	0.52
Antipyretic doses per hospital day, median [IQR]; mean (SD)	2.6 [0.9, 3.9]	2.5 [1.1, 3.9]	2.6 [0.9, 3.9]	0.87	3.1 (1.9)	3.5 (1.7)	0.46	2.4 [1.3, 3.6]	2.6 [0.7, 3.9]	0.51	1.1 [0.0, 3.0]	1.3 [0.3, 2.6]	0.83

[a] for cases with any oxygen supplementation
[b] details available as **supplementary data, Box S8**

Table S1 Baseline characteristics stratified by diagnostic group and discharge method; SD includes both pre- and post-implementation cohort

	Overall	All diagnoses		p-value	Bronchiolitis		p-value	Pneumonia		p-value	Obstructive Bronchitis / Asthma		p-value
**Number of cases**	352	352			120		P=	134			98		
**Method of discharge**	**All**	**CLD**	**SD**		**CLD**	**SD**		**CLD**	**SD**		**CLD**	**SD**	
**Number**	**352**	**62**	**290**		**22**	**98**		**25**	**109**		**15**	**83**	
Cases with readmission within fourteen days after discharge, n (%)	12 (3.4)	2 (3.2)	10 (3.4)	1	2 (9.1)	4 (4.1)	0.30	0 (0.0)	2 (1.8)	1	0 (0.0)	4 (4.8)	1
Cases with ED-visits within fourteen days after discharge, n (%)[a]	25 (7.1)	3 (4.8)	22 (7.6)	0.62	2 (9.1)	7 (7.1)	0.67	1 (4.0)	5 (4.6)	1	0 (0.0)	10 (12.0)	0.35
Cases with current minor complications / comorbidities, n (%)	103 (29.3)	15 (24.2)	88 (30.3)	0.42	7 (31.8)	22 (22.4)	0.51	5 (20.0)	49 (45.0)	0.02	3 (20.0)	17 (20.5)	1
Median time of day of discharge, HH:MM [IQR]	12:46 [11:20, 14:37]	13:14 [10:11, 15:34]	12:44 [11:24, 14:27]	0.64	13:00 [10:04, 14:53]	12:30 [11:30, 14:29]	0.87	13:02 [10:00, 15:44]	13:00 [11:57, 14:31]	0.65	14:50 [12:22, 15:38]	12:30 [11:00, 14:03]	0.08
Median length of hospital stay, days [IQR]	2.2 [1.5, 3.8]	2.0 [1.5, 3.3]	2.4 [1.5, 3.9]	0.45	2.3 [1.8, 4.9]	3.7 [1.8, 5.2]	0.50	2.0 [1.6, 2.9]	2.5 [1.6, 3.3]	0.35	1.6 [1.3, 2.7]	1.8 [1.3, 2.6]	0.92
Median duration of oxygen stop to discharge, hours [IQR][b]	22.8 [13.7, 28.6]	15.0 [7.4, 25.2]	24.0 [16.0, 28.9]	<0.001	18.1 [8.0, 24.6]	25.6 [18.0, 35.2]	0.02	16.1 [8.6, 26.4]	23.3 [18.4, 27.9]	0.04	9.9 [7.0, 22.6]	22.0 [14.4, 26.8]	0.03

[a] including subsequent readmissions
[b] Subanalysis for individuals that needed supplemental oxygen during hospitalisation

Table S2 Outcome table comparing CLD and SD cohorts, stratified by diagnostic group; SD includes both pre- and post-implementation cohort

Quality Group	Applicable groups combined		p-value	Bronchiolitis[a]		p-value	Pneumonia[a]		p-value	Obstructive Bronchitis / Asthma[a]		p-value
**Number of cases**	150			120		P=	134			98		
**Method of discharge**	**CLD[a]**	**SD**		**CLD[a]**	**SD**		**CLD[a]**	**SD**		**CLD[a]**	**SD**	
**Number**	individual	individual		**27**	**19**		**20**	**42**		**15**	**27**	
All discharge criteria fulfilled, n (%)	54 (87.1)	73 (83.0)	0.64	26 (96.3)	19 (100)	0.18	15 (75.0)	29 (69.0)	0.85	13 (86.7)	25 (92.6)	0.15
Sustained oxygen saturations of ≥ 90% for at least 6 hours, n (%)	62 (100.0)	88 (100.0)	NA	27 (100.0)	19 (100.0)	NA	20 (100.0)	42 (100.0)	NA	15 (100.0)	27 (100.0)	NA
No supplemental oxygen requirement for at least 6 hours, n (%)	61 (98.4)	87 (98.9)	1	26 (96.3)	19 (100.0)	1	20 (100.0)	41 (97.6)	1	15 (100.0)	27 (100.0)	NA
No respiratory distress at discharge, approximated with normal respiratory rate, n (%)	36 (85.7)	38 (82.6)	0.92	25 (92.6)	17 (89.5)	1				11 (73.3)	21 (77.8)	1
Patient afebrile (≤38.0°) for at least 24 hours, n (%)	15 (75.0)	29 (69.0)	0.05				15 (75.0)	29 (69.0)	0.05			
Inhalation interval stretched to at least 4-hourly, n (%)	13 (86.7)	25 (92.6)	0.61							13 (86.7)	25 (92.6)	0.61

[a]Quality group corresponds to in reality assigned discharge criteria groups for CLD cohorts and retrospectively assigned diagnostic groups for SD cohorts

Table S3: Fulfilment of disease specific discharge criteria, comparing post-implementation CLD and SD cohorts at discharge

Answers (20 total)<br>CLD only (18 total)<br>Question	Strongly disagree	Disagree	Neither nor	Agree	Strongly agree
**I felt that the moment of discharge was "just right".**	0 (0%)<br>0 (0%)	0 (0%)<br>0 (0%)	1 (5%)<br>1 (5.6%)	8 (40%)<br>6 (33.3%)	11 (55%)<br>11 (61.1%)
**I was happy with the communication concerning the timing of our/my discharge.**	1 (5%)<br>1 (5.6%)	0 (0%)<br>0 (0%)	0 (0%)<br>0 (0%)	7 (35%)<br>6 (33.3%)	12 (60%)<br>11 (61.1%)
**I knew what criteria were used to determine our discharge readiness.**	0 (0%)<br>0 (0%)	0 (0%)<br>0 (0%)	1 (5%)<br>1 (5.6%)	5 (25%)<br>3 (16.7%)	14 (70%)<br>14 (77.8%)
**My questions concerning the next few days, the care at home and contact persons in case of problems were satisfactorily answered.**	0 (0%)<br>0 (0%)	0 (0%)<br>0 (0%)	1 (5%)<br>1 (5.6%)	7 (35%)<br>6 (33.3%)	12 (60%)<br>11 (61.1%)
**I felt safe taking my child home / going home.**	0 (0%)<br>0 (0%)	0 (0%)<br>0 (0%)	0 (0%)<br>0 (0%)	6 (30%)<br>5 (27.8%)	14 (70%)<br>13 (72.2%)

Table S4: Survey results of caregiver / patient satisfaction with discharge

References

1. National Institute for Health and Care Excellence (NICE) (2021) Bronchiolitis in children: diagnosis and management. NICE guideline. National Institute for Health and Care Excellence (NICE), London, pp 1-26. https://www.ncbi.nlm.nih.gov/books/NBK573086/
2. Ralston SL, Lieberthal AS, Meissner HC, Alverson BK, Baley JE, Gadomski AM, Johnson DW, Light MJ, Maraqa NF, Mendonca EA, et al. (2014) Clinical practice guideline: the diagnosis, management, and prevention of bronchiolitis. Pediatrics 134:e1474-e1502. https://doi.org/10.1542/peds.2014-2742
3. World Health Organization (1990) Acute respiratory infections in children : case management in small hospitals in developing countries, a manual for doctors and other senior health workers., p 69. https://iris.who.int/handle/10665/61873
4. Gesellschaft für Pädiatrische Pneumologie, Deutsche Gesellschaft für Pädiatrische Infektiologie (2024) Management der ambulant erworbenen Pneumonie bei Kindern und Jugendlichen (paediatric community-Acquired pneumonia, pCAP), Aktualisierte Version 2.0 vom 25.01.2024. https://register.awmf.org/de/leitlinien/detail/048-013
5. Hayes D, Jr., Wilson KC, Krivchenia K, Hawkins SMM, Balfour-Lynn IM, Gozal D, Panitch HB, Splaingard ML, Rhein LM, Kurland G, et al. (2019) Home Oxygen Therapy for Children. An Official American Thoracic Society Clinical Practice Guideline. Am J Respir Crit Care Med 199:e5-e23. https://doi.org/10.1164/rccm.201812-2276ST
6. Principi T, Coates AL, Parkin PC, Stephens D, DaSilva Z, Schuh S (2016) Effect of Oxygen Desaturations on Subsequent Medical Visits in Infants Discharged From the Emergency Department With Bronchiolitis. JAMA Pediatr 170:602-608. https://doi.org/10.1001/jamapediatrics.2016.0114
